# Targeted therapy: resistance and re-sensitization

**DOI:** 10.1186/s40880-015-0047-1

**Published:** 2015-09-14

**Authors:** Dao-Hong Chen, Xiao-Shi Zhang

**Affiliations:** Biomedical Research Institute, Yiling Pharmaceutical Company, Beijing, 102600 P. R. China; Biotherapy Center, Sun Yat-sen University Cancer Center; State Key Laboratory of Oncology in South China; Collaborative Innovation Center for Cancer Medicine, Guangzhou, Guangdong 510060 P. R. China

**Keywords:** Targeted therapy, Drug resistance, Re-sensitization

## Abstract

The last two decades have witnessed a paradigm shift from cytotoxic drugs to targeted therapy in medical oncology and pharmaceutical innovation. Inspired by breakthroughs in molecular and cellular biology, a number of novel synthesized chemical compounds and recombinant antibodies have been developed to selectively target oncogenic signaling pathways in a broad array of tumor types. Although targeted therapeutic agents show impressive clinical efficacy and minimized adverse effects compared with traditional treatments, the challenging drug-resistant issue has also emerged to limit their benefits to cancer patients. In this regard, we aim to improve targeted therapy by presenting a systematic framework regarding the drug resistance mechanisms and alternative approaches to re-sensitize cancer cells/tissues therapeutically.

## Background

Anti-cancer drugs represent an efficacious weapon in the human defensive war against neoplastic diseases. As an ever-evolving force in fighting malignancies and improving patients’ quality of life, drug-therapeutic approaches have progressed through several milestones including cytotoxic compounds, hormonal treatments, targeted therapies, and combinational regimens. Anti-cancer drugs in the early period, such as 5-fluorouracil and methotrexate, were generally cytotoxic medicines without specific molecular targets. They usually killed rapidly dividing (cancer) cells by inhibiting DNA synthesis or related enzymes. Unfortunately, some normal cells, for example bone marrow and hair cells in the body, also divide very rapidly and were coincidently affected, resulting in severe adverse effects [1, 2]. Four decades ago, the rise of hormonal therapy, such as tamoxifen for breast cancer, has remarkably enhanced tissue selectivity and thereby diminished adverse systematic events [3]. However, the utility of hormonal therapy is limited to a few types of tumors with high sexual hormone activity as the driving force for growth.

During the last two decades, dramatic breakthroughs in cellular and molecular biology have resulted in the delineation of particular signaling pathways that control proliferation, cell death/differentiation, angiogenesis, and metabolism. As a result, this exciting scientific progress has been deeply inspiring innovative drug research and development, to undergo a fundamental transformation toward targeted therapy. To date, two categories of targeted therapeutic agents with unique properties in oncology, namely synthetic chemical compounds and recombinant monoclonal antibodies, are known to have achieved clinical successes [4, 5]. Most of the chemical compounds were designed to function as oncogenic kinase inhibitors that have good bioavailability, good tolerability, and few adverse effects. In contrast, therapeutic antibodies have prolonged half-lives in plasma, highly molecule-targeting specificity, and minimal off-target toxicity (if any).

Targeted therapy in the clinic is capable of delivering clear benefits to patients as evidenced by improvement in overall survival (OS), progression-free survival (PFS), and response rate (RR), among other end points. Effective anti-cancer treatments have so far been able to eliminate macroscopic tumors both at primary sites and at common distal sites. Nevertheless, even if they showed an impressive response to treatment initially, the majority of cancer patients relapsed because small cohorts of tumor cells can survive in cryptic anatomic loci and exhibit up to 90% resistance to one or more therapeutic agents for months or year(s). As such, drug resistance has been a major hurdle for classic anti-cancer medicines [1], and it still is a great challenge facing the emerged array of targeted therapies [2, 6]. Herein, we provide a comprehensive overview regarding targeted therapy resistance and alternative strategies in oncology (Table 1).

**Table 1:** Anti-cancer drugs for targeted therapy

Target	First-line drug(s)	Principal indication(s)	Potential resistance mechanism(s)	Re-sensitization agent(s)	References
BCR-ABL fusion	Imatinib	CML	Fixing the kinase domain in its active configuration	1. Dasatinib 2. Nilotinib 3. Ponatinib	[7, 8]
KIT	Imatinib	GIST Melanoma Sarcoma	1. Mutations of *c*-*KIT* axons 9/11 2. *PDGFRA* (D842 V) mutation	1. Sunitinib 2. Regorafenib	[7, 8]
EGFR	Gefitinib Erlotinib Cetuximab	NSCLC Colorectal cancer	1. Secondary mutations in *EGFR* such as T790 M “gatekeeper” mutation 2. Multi-signaling cascade-driven disorders 3. *EGFR* S492R mutation	1. Cetuximab + Afatinid 2. Rociletinib 3. Panitumumab	[9–13]
ALK rearrangement	Crizotinib	NSCLC	Secondary mutations in *ALK* rearrangement	Ceritinib Alertinib	[14, 15]
HER2	Trastuzumab	Breast cancer Gastric cancer	1. Lack of the extracellular domain of HER2 2. Enhanced Her2 dimerization	1. Pertuzumab 2. Herceptin emtansine 3. Lapatinib	[16, 17]
VEGF/VEGFR	Bevacizumab Ramucirumab Sorafenib Sunitinib Aflibercept Pazopanib Vandetanib Vatalanib Cediranib Axitinib	Renal cancer Colorectal cancer Small cell lung cancer Thyroid cancer Sarcoma	1. Alternative angiogenic signaling 2. Increased HIF1, MDSCs, CSCs, etc.	1. Alternative angiogenic inhibitors 2. CSC inhibitors	[18–21]
BRAF	Vemurafenib Dabrafenib	Melanoma	1. BRAF amplification and new BRAF V600E splice isoforms 2. BRAF-independent MAPK activation	1. Vemurafenib + Cobimetinib 2. Dabrafenib + Trametinib 3. Anti-PD-1/PD-L1 antibodies	[22–24]

*BCR*-*ABL* breakpoint cluster region-Abelson, *CML* chronic myeloid leukemia, *KIT* kit receptor tyrosine kinase, *GIST* gastrointestinal stromal tumor, *PDGFRA* platelet-derived growth factor receptor A, *EGFR* epidermal growth factor receptor, *NSCLC* non–small cell lung cancer, *ALK* anaplastic lymphoma kinase, *HER2* human EGFR2, *VEGF(R)* vascular endothelial cell growth factor (receptor), *HIF1* hypoxia-inducible factor 1, *MDSC* myeloid-derived suppressor cell, *CSC* cancer stem cell, *BRAF* raf murine sarcoma viral oncogene homolog B, *MAPK* mitogen-activated protein kinase, *PD*-*1* programmed cell death protein 1, *PD*-*L1* programmed death-ligand 1

## Breakpoint cluster region-Abelson (*BCR*-*ABL*) inhibitor

Imatinib (Gleevec, Novartis, Basel, Switzerland) revolutionized therapeutic strategies for chronic myeloid leukemia (CML) through targeting the BCR-ABL fusion protein, which drives constitutive tyrosine kinase activity and in turn the malignant behaviors of leukemic cells [7]. Impressively, imatinib has been able to extend 5-year OS rate of CML patients beyond traditional treatments, thereby re-defining CML as a manageable disease [25]. Additionally, imatinib is also a potent antagonist of c-kit receptor tyrosine kinase (c-KIT*)* and platelet-derived growth factor receptor A (PDGFRA) kinase, both of which cause gastrointestinal stromal tumors (GIST) [26]. In the pharmaceutical industry, the success of imatinib evoked a huge wave of efforts to develop various disease-associated kinase inhibitors. However, as an era of targeted therapy comes following the light of the first BCR-ABL inhibitor, resistance to imatinib is emerging as a major challenge in CML management.

Imatinib resistance results from complicated mechanisms including up-regulated multidrug resistance (MDR) proteins. However, mutations (such as T315I) in the *BCR*-*ABL* gene were revealed to be the most common mechanism behind imatinib resistance, and they associate with an advanced disease state (accelerated or blast-phase CML). Imatinib works as an adenosine triphosphate (ATP) mimetic compound, and it only binds to the inactive conformation of the enzyme. Mutations of *BCR*-*ABL* that fix the kinase domain in its active configuration result in diminished binding to the compound and, therefore, a loss of inhibitory potency. To address imatinib resistance issue in CML, new-generation inhibitors, such as dasatinib, nilotinib, and ponatinib, were developed to suppress the enzyme with a capability of potently binding its active conformation [7]. Likewise, in the case of GIST, imatinib resistance mainly results from mutations of the c-*KIT* and *PDGFRA* genes. Primary resistance in GIST occurs in 6 months of drug treatment, and it is due to mutations in catalytic domain of c-*KIT* (exon 9) or *PDGFRA* (D842V). Moreover, secondary resistance to imatinib appears approximately 2 years after the treatment, and it is associated with alternative c-*KIT* mutations such as V654A and N822K plus exon 11 mutations. In response to these challenges, sunitinib and regorafenib have been developed to serve as second- and third-generation inhibitors, respectively, for GIST treatment [2, 7, 8].

## Inhibitors of epidermal growth factor receptor (EGFR) and anaplastic lymphoma kinase (ALK)

EGFR represents a member of the cell surface receptor tyrosine kinase (RTK) molecular family, and it is activated upon ligand binding as well as receptor dimerization. The activation of EGFR and its down-stream pathways, such as extracellular receptor kinase (ERK) and protein kinase B (AKT), substantially contributes to cell proliferation, survival, migration, and angiogenesis. Up-regulation of EGFR signaling activity occurs in many types of cancers and is thus an attractive target for contemporary drug development [27]. EGFR inhibitors that are currently available include gefitinib, erlotinib, monoclonal antibody cetuximab, and others [9]. Being less toxic, gefitinib and erlotinib have been reported to be superior to conventional cytotoxic chemotherapy in terms of RR and PFS time in lung adenocarcinoma patients with *EGFR* mutations such as L858R (*EGFR* addiction). In addition, cetuximab in combination with radiation in head and neck cancer has delivered more impressive benefits, increasing the 2-year OS rate of the patients [13]. Additionally, cetuximab was approved for treating metastatic and chemotherapy-resistant colorectal cancer due to its clinical efficacy with improved PFS and RR [10, 13].

Not all EGFR-expressing cancers respond to targeted inhibitor treatment. Moreover, those patients that benefit from EGFR inhibitors beyond conventional chemotherapy initially become resistant to the targeted therapy inevitably after approximately 1 year. The most common mechanism of primary and acquired resistance to EGFR inhibitor in lung cancer is the T790 M “gatekeeper” mutation, for which a currently available solution is combining cetuximab with afatinid. However, an *EGFR* mutation S492R in colorectal cancer leads to resistance to cetuximab, which can be overcome by the newer EGFR antibody panitumumab. Meanwhile, it is anticipated that EGFR inhibitors of second- or third-generation will be coming out to overcome target-resistant cancers. Of note, most malignancies with high EGFR expression can be multi-signaling cascade-driven disorders under certain circumstances. Depending on the cellular/molecular contexts, many other compensatory pathways, such as *Ras/*phosphatidylinositol-3-kinases (*PI3K*) mutations and MET/human epidermal growth factor receptor 2 (HER2) high activity, have also been found to contribute to EGFR inhibitor resistance [11, 12]. Accordingly, a combination of EGFR with PI3K or MET pathway inhibitors appears necessary in this case.

Interestingly, a chromosomal translocation-induced ALK activation has been identified in 3–5% of non–small cell lung cancer (NSCLC) patients, and is clinically linked to a more aggressive phenotype. This subset of lung cancer is addicted to high ALK activity for malignant growth, and in general it is dramatically sensitive to ALK-targeted agents such as crizotinib. Although the ALK inhibitor was able to significantly improve the clinical outcome of NSCLC patients with a RR of 60% and a PFS of 10 months, the emergence of resistance to crizotinib has been noticed as a challenge [14, 15]. Similar to the scenarios in the above therapeutic targets, the *ALK* gene mutations such as C1156Y and L1196M have been reported as the molecular mode of crizotinib resistance [14]. Moreover, alternative signaling activation, including EGFR and c-KIT, was proposed to be a minor mechanism [2]. To address this issue, ALK inhibitors of new generation, such as LDK378 and AP26131, are currently in clinical trials and have shown encouraging efficacy [15].

## Human EGFR2 (HER2) antibody

Breast cancer consists of a heterogeneous mass of pathologic conditions which, for therapeutic guidance purposes, are classified as hormone receptor–positive, human EGFR2–positive (HER2^+^), or triple-negative groups. Strategies targeting the estrogen receptor (ER) and HER2 to treat breast cancer have come up with some of the most successful drugs in oncology. HER2 is overexpressed in 25–30% of breast cancer patients, predicting a poor clinical outcome in the absence of targeted therapy [27, 28]. Trastuzumab (Herceptin) is a humanized monoclonal antibody targeting the extracellular domain of the HER2 protein, and trastuzumab plus chemotherapy has been accepted as the standard first-line treatment for HER2-positive metastatic breast cancer (MBC). Application of trastuzumab was revealed to confer extra-clinical benefits to HER2-positive breast patients with significantly longer PFS, higher RR, and reduced relapse compared with conventional therapy [29]. Even if the cancer progresses on trastuzumab, the anti-cancer drug is still recommended for continuation due to its proven clinical efficacy on slowing tumor growth [29, 30].

The majority of HER2-positive breast cancer patients would not respond to trastuzumab without simultaneous cytotoxic therapy. It has been proposed that trastuzumab exerts clinical efficacy mainly through sensitizing tumor cells to DNA damage by cytotoxic chemotherapy [2, 30]. Notably, a truncated 95–100 kDa receptor (p95HER2), expressed in a minor portion of HER2-positive breast cancer patients, leads to an aggressive trastuzumab-resistant phenotype due to its lack of the extracellular domain of HER2 targeted by trastuzumab [16]. Moreover, enhanced HER2 dimerization resulting from *PI3* *K* mutations and/or loss of phosphatase and tensin homolog (*PTEN*) was identified to play an important role in secondary resistance to trastuzumab [2, 27]. To circumvent these problems, pertuzumab, a newer HER2 antibody, has been developed to disturb HER2/HER3 dimerization, thereby augmenting trastuzumab activity [17]. In addition, trastuzumab emtansine, derived from covalently conjugating a cytotoxic compound with the antibody, retains therapeutic activity in patients resistant to trastuzumab. Moreover, lapatinib, a reversible inhibitor of EGFR and HER2, has been approved to treat HER2-positive breast cancer upon trastuzumab failure [28].

## Vascular endothelial growth factor (VEGF) antagonists

The VEGF to its receptor (VEGFR) signaling plays a pivotal role in new blood vessel formation termed angiogenesis, and this activity is significantly up-regulated in solid tumor tissues upon growth beyond 1 mm in size. In corollary, approaches targeting tumor angiogenesis represent an exciting option in anti-cancer therapy through modulating the microenvironment [31]. Bevacizumab (Avastin), a humanized VEGF-A neutralizing antibody, represents the first-generation agent of anti-tumor angiogenesis and has been approved to treat several types of malignancies in combination with cytotoxic chemotherapy, including colon, lung, and renal cancers [32]. Later on, inspired by this idea, more VEGF/VEGFR antagonists, including decoy receptors and small chemical inhibitors, were developed and have also achieved clinical success [2]. Interestingly, the VEGFR inhibitors sunitinib and vandetanib coincidentally inhibit c-*KIT* and rearranged during transfection (*RET*) genes, thereby having additional benefits for treating GIST and medullary thyroid carcinoma, respectively [18]. After showing dramatic anti-tumor efficacy in animal models, various angiogenesis inhibitors have so far conferred moderate improvements in PFS, RR, and OS in patients with certain cancer types in the clinic [18, 19].

Even though mutations of receptor or kinase domain genes have been identified as the major reason of resistance to other targeted therapies such as ACR-ABL and EGFR inhibitors, this is not the case in VEGF-targeted drug resistance. The mechanisms behind resistance to VEGF antagonists are complex and have not been completely understood yet. Notably, there are a variety of alternative signaling molecules, such as placental growth factor (PIGF), fibroblast growth factor (FGF), and angiopoietin, which can bypass VEGF/VEGFR inhibition and restore tumor angiogenesis. In this regard, adding inhibitors against these alternative angiogenesis targets to therapeutic programs has been proposed to be helpful for overcoming anti-VEGF resistance [2, 19]. Furthermore, continued inhibition of angiogenesis creates a hypoxic environment and in turn induces secondary pathologic alterations in tumor tissues, including increased hypoxia inducible factor 1 α (HIF1α), myeloid-derived suppressor cells (MDSCs), and cancer stem cells (CSCs). As a transcription factor, HIF1α up-regulates a wide array of genes associated with cell survival and anaerobic metabolism [20]. Meanwhile, cancer and stromal cells secrete granulocyte colony-stimulating factor (GCSF) and thus activate MDSCs which also contribute to drug resistance via suppressing immune activity against cancer in the tumor microenvironment. Recently, CSCs have been recognized as a particular subpopulation of cancer cells that are responsible for metastasis and therapeutic resistance [33]. In this sense, to solve anti-VEGF drug resistance problems in the long-term, the hypoxia-induced secondary pathologic changes need to be reversed by combining with certain therapeutic means accordingly.

## Raf murine sarcoma viral oncogene homolog B (BRAF) inhibitor

The serine/threonine kinase BRAF serves as a crucial mediator in rat sarcoma (RAS) mitogen-activated protein kinase (MAPK) signaling cascade which represents a defined pathway driving cell growth and becomes an oncogene upon mutation. To date, *BRAF* mutations have been identified in many types of tumors, and are highly frequent in melanoma, colorectal cancer, and papillary thyroid cancer. Over 50% of melanomas bear *BRAF* mutations at the advanced stage, of which approximately 80% appear as the substitution of glutamic acid (E) for valine (V) in codon 600, known as the V600E mutation, and approximately 16% as the V600K mutation. To circumvent these molecular targets, vemurafenib, a highly selective inhibitor of *BRAF* V600E mutation, was developed and have shown clear clinical benefits including significantly better response and higher OS rates compared with commonly utilized standard chemotherapy agents [22]. Recently, another *BRAF* mutation inhibitor, dabrafenib, has been approved by the United States Food and Drug Administration (FDA) based on its clinical benefits of better PFS and RR. Excitingly, these two compounds represent the first drugs showing clinical benefits in patients with melanoma metastasis to the brain [23].

In spite of an impressive RR of 50% with vemurafenib and dabrafenib initially, acquired resistance to these drugs occurs after approximately 6 months. Distinct from the mechanisms in other tumors, secondary mutations of the target gene in melanomas have not been found. Instead, *BRAF* amplification and new *BRAF* V600E splice isoforms appear to account for the drug resistance [24]. Another possibility is BRAF-independent MAPK activation due to Ras bypass signaling or *MEK* mutations [2]. In this light, combining the MEK inhibitor trametinib with BRAF inhibitors in melanoma treatments has resulted better clinical outcomes than either therapy alone [23, 24]. Interestingly, the blocking antibodies (ipilimumab and nivolumab) of co-suppressing molecules cytotoxic T-lymphocyte-associated protein 4 (CTLA-4) and programmed cell death protein 1 (PD-1)/programmed death-ligand 1 (PD-L1) in immune cells have emerged to be quite promising in treating immunity-associated malignancies such as melanoma. To date, these immune cell–modulating antibodies added to therapeutic programs for melanomas have shown greater clinical efficacy and improved toxic profiles [34]. Impressively, the therapeutic response duration with nivolumab has been extended beyond 2 years [23].

## Conclusion and perspective

As a contemporary hallmark of medical oncology and innovation in pharmaceutical industry, targeted therapy provides physicians and patients with more options to improve clinical outcomes. Although imatinib has transformed CML into a manageable medical condition like other non-malignant diseases in several ways [25], the clinical benefits from most targeted therapies are limited, extending PFS or OS of cancer patients by only a few months, and resistance is eventually faced, followed by tumor aggression. There has been a consensus that, to improve the results of targeted therapy, challenging drug resistance problems needs to be solved through better understanding the mechanisms.

Unlike cytotoxic medicines in which modes of resistance are mainly attributed to dysregulated pharmacokinetic processes such as increased drug efflux out of responding cells mediated via *MDR* gene-encoded protein transporters [35], resistance to targeted therapy in oncology usually results from target gene mutation(s) and redundant activation of other pro-survival signaling pathways. In this sense, combination therapy with forthcoming next-generation agents targeting resistance-associated mutations and pathways appears to be a helpful strategy to circumvent the challenge. To do so smartly, it is necessary to note that selective biomarkers have come up with predictive information concerning the therapeutic sensitivity [36, 37] and enhanced the likelihood of right drug(s) for each individual patient which is regarded as personalized medicine. Furthermore, cancer genome monitoring and microRNA profiling [38] are being actively translated from bench to bedside in oncology, to hopefully help therapeutic selection and adjustment.

We should not ignore certain emerging scientific concepts with translational potential, such as CSCs which are speculated as the “root” for malignant recurrence and metastasis, being often linked to drug resistance. A variety of CSC inhibitors, either novel agents or old compounds from therapeutic repositioning, are increasingly available and able to improve clinical outcomes [21, 33]. In addition, the renaissance of cancer immunology in recent years has dramatically inspired oncology with several immune co-regulator targeting antibodies that deliver impressive clinical benefits [34], including extending the therapeutic response time to over 2 years [23]. Thus, it is conceivable that the body’s defense system holds deep resources to be explored to improve clinical outcomes such as overcoming drug resistance in oncology.
